# Supplementary material for: Shaping of inner ear sensory organs through antagonistic interactions between Notch signalling and Lmx1a
Source: eLife. 2017 Dec 4;6:e33323. doi: 10.7554/eLife.33323 (PMC5724992; doi:10.7554/eLife.33323)
Supplement: Transparent reporting form [file elife-33323-transrepform.docx]

***eLife’s* transparent reporting form**

We encourage authors to provide detailed information *within their submission* to facilitate the interpretation and replication of experiments. If you have any questions, please contact us: [editorial@elifesciences.org](mailto:editorial@elifesciences.org).

**Sample-size estimation**

- You should state whether an appropriate sample size was computed when the study was being designed
- You should state the statistical method of sample size computation and any required assumptions
- If no explicit power analysis was used, you should describe how you decided what sample (replicate) size (number) to use

Please outline where this information can be found within the submission (e.g., page numbers or figure legends), or explain why this information doesn’t apply to your submission:

For the current study, we chose to include a minimum of 6 biological replicates for each experimental paradigm (Methods, page 17). A biological replicate refers to samples obtained from different embryos. For experiments where there was large variation within the data set, the number of replicates was increased (see results section page 5).

No statistical tests were used in the analysis due to the nature of the data. The data included in the current study are mainly qualitative data describing differences in gross inner ear morphology or the presence or absence of structures/cellular markers.

**Replicates**

- You should report how often each experiment was performed
- You should include a definition of biological versus technical replication
- The data obtained should be provided and sufficient information should be provided to indicate the number of independent biological and/or technical replicates
- If you encountered any outliers, you should describe how these were handled
- Criteria for exclusion/inclusion of data should be clearly stated
- High-throughput sequence data should be uploaded before submission, with a private link for reviewers provided (these are available from both GEO and ArrayExpress)

Please outline where this information can be found within the submission (e.g., page numbers or figure legends), or explain why this information doesn’t apply to your submission:

The number of experimental replicates used for each experiment in this study was chosen based on the variability of results.

For example in the Delta-1 over expression experiments, the results were variable. We therefore examined the effects of Delta-1 over expression in 21 biological replicates (page 5, bottom and Figure 3).

For the remaining experiments: Lineage tracing experiments (Figure 1 and S1) NICD over expression (Figure 4); cLmx1b over expression (Figure 5 & 6) and analysis of Lmx1a null tissue (Figure 8, 9 & 10) the results were very consistent. For these experiments we therefore used a minimum of 6 biological replicates for each paradigm (see Methods, page 17).

Exclusion criteria for samples were based on absence of fluorescence following electroporation. If no fluorescence was observed, it was assumed that transfection had not been successful.

All samples in which fluorescence was clearly seen in the inner ear were included in the analysis.

**Statistical reporting**

- Statistical analysis methods should be described and justified
- Raw data should be presented in figures whenever informative to do so (typically when N per group is less than 10)
- For each experiment, you should identify the statistical tests used, exact values of N, definitions of center, methods of multiple test correction, and dispersion and precision measures (e.g., mean, median, SD, SEM, confidence intervals; and, for the major substantive results, a measure of effect size (e.g., Pearson's r, Cohen's d)
- Report exact p-values wherever possible alongside the summary statistics and 95% confidence intervals. These should be reported for all key questions and not only when the p-value is less than 0.05.

N/A

Please outline where this information can be found within the submission (e.g., page numbers or figure legends), or explain why this information doesn’t apply to your submission:

(For large datasets, or papers with a very large number of statistical tests, you may upload a single table file with tests, Ns, etc., with reference to page numbers in the manuscript.)

**Additional data files (“source data”)**

- We encourage you to upload relevant additional data files, such as numerical data that are represented as a graph in a figure, or as a summary table
- Where provided, these should be in the most useful format, and they can be uploaded as “Source data” files linked to a main figure or table
- Include model definition files including the full list of parameters used
- Include code used for data analysis (e.g., R, MatLab)
- Avoid stating that data files are “available upon request”

Please indicate the figures or tables for which source data files have been provided:

N/A
